# Comparative transcriptional profiling analysis of the two daughter cells from tobacco zygote reveals the transcriptome differences in the apical and basal cells

**DOI:** 10.1186/1471-2229-10-167

**Published:** 2010-08-11

**Authors:** Tian-Xiang Hu, Miao Yu, Jie Zhao

**Affiliations:** 1 Key Laboratory of the Ministry of Education for Plant Developmental Biology, College of Life Sciences, Wuhan University, Wuhan 430072, China

## Abstract

**Background:**

In angiosperm, after the first asymmetric zygotic cell division, the apical and basal daughter cells follow distinct development pathways. Global transcriptome analysis of these two cells is essential in understanding their developmental differences. However, because of the difficulty to isolate the *in vivo *apical and basal cells of two-celled proembryo from ovule and ovary in higher plants, the transcriptome analysis of them hasn't been reported.

**Results:**

In this study, we developed a procedure for isolating the *in vivo *apical and basal cells of the two-celled proembryo from tobacco (*Nicotiana tabacum*), and then performed a comparative transcriptome analysis of the two cells by suppression subtractive hybridization (SSH) combined with macroarray screening. After sequencing, we identified 797 differentially expressed ESTs corresponding to 299 unigenes. Library sequence analysis successfully identified tobacco homologies of genes involved in embryogenesis and seed development. By quantitative real-time PCR, we validated the differential expression of 40 genes, with 6 transcripts of them specifically expressed in the apical or basal cell. Expression analysis also revealed some transcripts displayed cell specific activation in one of the daughter cells after zygote division. These differential expressions were further validated by *in situ *hybridization (*ISH*). Tissue expression pattern analysis also revealed some potential roles of these candidate genes in development.

**Conclusions:**

The results show that some differential or specific transcripts in the apical and basal cells of two-celled proembryo were successfully isolated, and the identification of these transcripts reveals that these two daughter cells possess distinct transcriptional profiles after zygote division. Further functional work on these differentially or specifically expressed genes will promote the elucidation of molecular mechanism controlling early embryogenesis.

## Background

Embryo development from one-celled zygote to mature embryo is a critical part of the life cycle in higher plants. During double fertilization, one sperm cell from pollen grain fuses with an egg cell from embryo sac, and the resultant zygote undergoes a series of precise cell divisions and develops into an embryo [1,2]. In most angiosperms, the first zygotic cell division is transverse and asymmetric, resulting in the formation of a two-celled proembryo with a small apical cell and a large basal cell. The small apical cell with dense cytoplasm develops into embryo proper, and the large vacuolated basal cell differentiates into hypophysis and suspensor. The hypophysis contributes to the formation of root meristem within the embryo proper [3]. The suspensor, a terminally differentiated embryonic region, connects the embryo proper to the surrounding maternal tissues, serves as a conduit for nutrients and growth regulators supporting embryo development, and degenerates in the late embryo development [4]. In *Arabidopsis*, the mutations of *gnom *(*gn*), *root-shoot-hypocotyl-defective *(*rsh*) and *yoda *(*yda*) alter the asymmetric division of zygote, and result in the formation of two nearly equal-sized daughter cells and subsequent defect of embryonic axis establishment [5-7]. It suggests that the asymmetric division of zygote producing the apical and basal cells is a crucial event of early embryogenesis.

Previous researchers adopted various techniques and experiment systems to investigate embryogenesis mechanism. In lower plant, the zygote and embryo of brown alga (*Fucus*) have long been served as a cellular model to investigate early embryogenesis because of their development free of maternal tissue [8-10]. However, embryo sac in higher plants is typically surrounded by the sporophytic tissues of ovule and ovary, thus access to the embryo is hampered. To overcome these difficulties, the researchers utilize some *in vitro *culture systems to study the early embryo development mechanism [11-15]. Compared with embryogenesis *in vivo*, there are some differences in the way of embryos originate and develop, therefore, the results obtained *in vitro *fail to explain all the questions.

Since specific gene expression is usually linked directly to different developmental process, many techniques are exploited to identify genes expressed in the developing embryo, including cDNA library construction [16], promoter/enhancer trapping [17] and mutational screens [18,19]. Several embryo essential genes, such as *gn*, *twin *(*twn*), *monopteros *(*mp*), *bodenlos *(*bdl*), *topless *(*tpl*) and *yda*, were successfully identified by the mutant analysis in *Arabidopsis *[6,7,20-23]. cDNA libraries from complex tissues such as ovule are not efficient in identifying genes expressed at low level or only in the early several-celled proembryos. Recently, the development of laser capture microdissection (LCM) makes it possible to analyze the transcriptional profiles in specific embryo domains [24,25], but the single egg cell, zygote or early several-celled embryo are still too small to be isolated. Fortunately, micromanipulation, a powerful skill, is used successfully to isolate single cells from the embryo sac of some species such as maize, barley, tobacco, wheat and rice [11,26-29]. This technique combined with the transcriptome assay broadens our knowledge of gene expression in egg cell, central cell, zygote and proembryo [30-34], and these valuable information help us to understand certain critical questions such us zygote gene activation in higher plants.

Some genes up- or down-regulated in the two daughter cells from *in vitro *fertilized maize zygote were identified by Okamoto *et al. *[35]. However, besides the difference of embryogenesis *in vitro *and *in vivo*, there are even greater differences between embryo development in monocotyledon and dicotyledon plants. In contrast with the fixed and traceable division pattern during early embryogenesis in classic dicotyledon plants, variant cell division occurs during the proembryo development of monocotyledon plants [36]. Up to now, the analysis of transcriptional profiles in the *in vivo *apical and basal cells from the two-celled proembryo in dicotyledon plants is not reported. Therefore, in the present study, we focused on the transcriptome differences between the two cells in dicotyledonous plant tobacco. We originally established a procedure for isolating the live apical and basal cells of the *in vivo *two-celled proembryo just after the first cell division of zygote, and then the SMART PCR synthesized cDNAs from these two cells were used for SSH analysis. After macroarray screening and sequencing of the candidate clones, we successfully identified 797 ESTs that were specifically or predominantly expressed in the apical or basal cells. The ESTs were further analyzed, including comparative studies on the different transcript composition, functional classification, and validation by real-time PCR and *in situ *hybridization in the zygote and its apical and basal daughter cells. Also, the expression patterns of some identified ESTs were analyzed in different organs and tissues. The transcriptional composition differences in the apical and basal cells and possible function of some candidate differential transcripts are further discussed.

## Results

### Establishment of isolation procedures for the apical and basal cells from the two-celled proembryo

**Figure 1:**
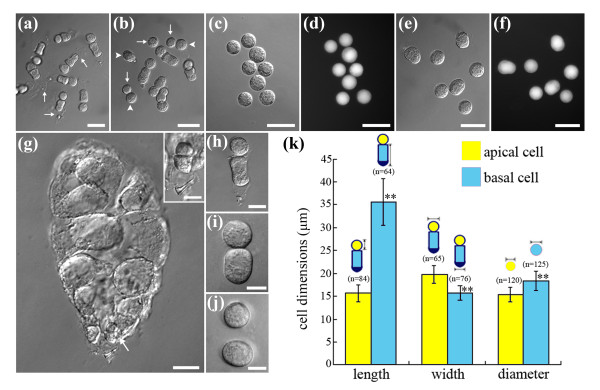
**Isolation of the two-celled proembryos and protoplasts of the apical and basal cells in tobacco**. (a) Freshly isolated two-celled proembryos with cell wall (arrows). (b) Two attached protoplasts derived from cell wall-digested two-celled proembryo. The small (arrows) and large protoplasts (arrowheads) are derived from the apical and basal cells of the two-celled proembryos, respectively. (c) Small living protoplasts from the apical cells. (d) Fluorescent image of the same protoplasts in (c), stained with FDA. (e) Large living protoplasts from the basal cells. (f) Fluorescent image of the same protoplasts in (e), stained with FDA. (g) One fertilized embryo sac with a two-celled proembryo (arrow) and a lot of endosperm cells. Inset is a magnification of the two-celled proembryo in embryo sac. (h-j) The detailed isolation process of a pair of protoplasts from a two-celled proembryo in an individual droplet. (k) Cell size of the apical and basal cells, and diameter of the protoplast from the *in vivo *two-celled proembryos. Bars in (k) show the mean ± standard error (SE), and "n" means the number of measured cells. Bar = 30 μm in Figure a-g; Bar = 15 μm in Figure h and inset; Bar = 10 μm in Figure i and j. **Significant difference at P < 0.01.

A well established method based on enzymatic maceration combined with brief micromanipulation [12] allowed us to isolate enough two-celled proembryos (Figure 1a, g, h). To study the transcriptome differences of the apical and basal cells, we established a procedure to get the separated protoplasts. Based on the enzyme mixture from Okamoto *et al. *[35], we optimized the key factors including incubation temperature and time for enzyme treatment of the two-celled proembryos to separate the apical and basal cells and preserve their viability under sterile environment. Incubation in 25°C for 15 min was tested to be the most suitable conditions for the isolation of the two cells. To avoid confusing the apical and basal cells from different proembryos, each proembryo was digested in an individual droplet of enzyme solution. The intact isolation process was shown in Figure 1g-j. After incubation, a pair of living protoplasts from the small apical cell and the large basal cell (Figure 1i, j) was isolated and then collected respectively (Figure 1c, e). The bright fluorescein diacetate (FDA) fluorescence emitted from the protoplasts indicated their strong viability (Figure 1d, f). Two transcription inhibitors, actinomycin D and cordycepin were added in the solutions to inhibit gene expression in response to possible stresses during the isolation process.

After the first zygotic division, tobacco zygote produces two asymmetric daughter cells just like most classic dicotyledonous plants. Morphologic observation of the isolated two-celled proembryoes revealed that the shape and size of apical cell is distinct from those of the basal cell. As shown in Figure 1h, the apical cell is small and nearly spherical, whereas the basal cell is relatively large and elongated. To epitomize the cell size differences between the two cells, we measured cell length in vertical axis, width in transverse axis as well as the diameters of the two protoplasts (Figure 1k). The results show that the ratios of the basal cell to the apical cell are 2.28 in length and 0.80 in width, and the diameter of basal cell protoplast is larger than that of apical cell, with 18.37 ± 2.05 μm versus 15.41 ± 1.54 μm. To display the distinct difference in size, several couples of the isolated apical and basal cells were shown in the same bright field (Figure 1b).

### cDNA synthesis and identification of differentially expressed genes between the apical and basal cells

**Figure 2:**
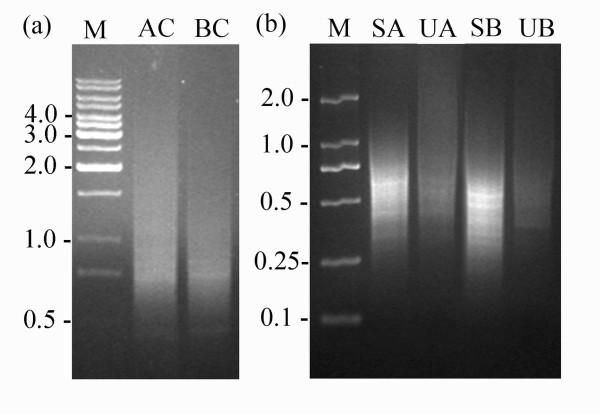
**Gel electrophoresis images of cDNA from the apical and basal cells of tobacco two-celled proembryo by LD-PCR and products of PCR-select cDNA subtraction**. (a) cDNAs synthesized from the apical cell (AC) and basal cell (BC). M, Molecular marker 1 kb DNA ladder. (b) Products of PCR-select cDNA subtraction. M, Molecular marker DL2000; SA, the second PCR product of the apical cell/basal cell subtraction; UA, the second PCR product of the unsubtracted control; SB, the second PCR product of the basal cell/apical cell subtraction; UB, the second PCR product of the unsubtracted control.

About three hundred pairs of apical and basal cells from the two-celled proembryos were isolated and collected respectively for RNA isolation. The first-strand cDNA was synthesized by applying a template-switch mechanism to the 5'end of the RNA template (SMART) during reverse transcription and then amplified by long-distance PCR (LD-PCR). After the optimization of PCR cycle number, all amplification of sample cDNA was then carried out for 23 PCR cycles. Generally, the final cDNA products from both types of cells mainly distributed from 0.5 kb to 4 kb in gel electrophoresis, with nearly equal distribution over the whole size range (Figure 2a).

To reveal the transcriptome differences of the apical and basal cells, suppression subtractive hybridization (SSH) was applied to identify the differentially expressed genes. Both forward (apical cell/basal cell, apical cell cDNA as tester) and reverse (basal cell/apical cell, basal cell cDNA as tester) subtracted cDNA libraries were constructed to enrich the genes specifically or predominantly expressed in the apical or basal cells. Pools of putative differentially expressed cDNA were obtained after two rounds of subtraction. Compared with their respective unsubtracted control, both the subtracted DNA samples displayed a quite different distribution with a number of distinct bands. The forward subtracted cDNA ranged mainly from ~300 bp to ~1 kb and the reverse subtracted cDNA from ~250 bp to ~750 bp (Figure 2b).

### Bioinformatic analysis of ESTs

After two rounds of differential screening, the candidate clones were selected for sequencing. Then a total of 797 ESTs sequences, 385 for the apical cell (with library ID AC001C- 385C) and 412 for the basal cell (with library ID BC001C-412C) were generated. The 797 sequences were clustered and assembled into 91 contiguous sequences (contigs; Additional file 1) and 208 single sequences (singletons), with 43 contigs (with library ID ACC01-43) and 124 singletons from the apical cell, and 48 contigs (with library ID BCC01-48) and 84 singletons from the basal cell. Therefore, these 797 ESTs represented 299 unique transcripts. Further BLASTX analysis showed that 131 apical cell transcripts (78.4% of apical cell transcripts) and 78 basal cell transcripts (59.5% of basal cell transcripts) matched significantly (*E*-value < 10^-5^) to database entries with assigned identities, which mainly generated from different tissues of grape, tomato, *Arabidopsis*, tobacco and rice. The 91 apical and basal cell contigs that consist of two or more ESTs are respectively shown in Table 1a and 1b, and the detailed blast annotations of all unigenes are listed in Additional file 2.

**Table 1:** Functional annotation of the differentially expressed contigs with two or more ESTs between the apical cells (a) and basal cells (b)

ID	EST Number	BLASTX sequence similarity (^a^BlastN)	Organism	Accession	E-Value
(a) Apical cell					
ACC01	32	hypothetical protein	R. communis	XP_002531967	4.95E-14
ACC02	32	unknown protein	G. max	ACU24256	1.07E-19
ACC03	30	hypothetical protein	V. vinifera	CAN70790	1.53E-25
ACC04	17	hypothetical protein	N. tabacum	YP_173374	2.03E-35
ACC05	14	cytochrome P450 like protein	N. tabacum	BAA10929	2.32E-58
ACC06	14	histone h3	Z. mays	ACG25088	4.31E-27
ACC07	10	histone h2a	N. tabacum	BAC53941	3.89E-39
ACC08	8	40s ribosomal protein s23	S. tuberosum	ABB16993	1.83E-76
ACC09	7	histone h2a	N. tabacum	BAC53941	7.74E-38
ACC10	6	histone h2a	S. melongena	BAA85117	8.24E-26
ACC11	5	b-type cyclin	R. communis	XP_002530166	3.21E-14
ACC12	5	lipid transfer proteins related	V. vinifera	XP_002281585	9.59E-47
ACC13	4	dehydrin	N. tabacum	BAD13499	2.52E-27
ACC14	4	histone h3	B. floridae	XP_002595193	4.18E-26
ACC15	4	histone h4	E. japonica	ACX50406	1.71E-38
ACC16	4	atp-dependent helicase	V. vinifera	XP_002277541	7.63E-24
ACC17	3	3'-5' exonuclease	V. vinifera	XP_002277523	1.15E-46
ACC18	3	calmodulin	S. commersonii	P27161	8.15E-78
ACC19	3	cyclophilin	C. annuum	ACB05668	7.70E-48
ACC20	3	histone h2a	S. melongena	BAA85117	3.49E-25
ACC21	3	histone h3	M. pusilla	EEH57511	7.14E-14
ACC22	3	histone h4	P. sitchensis	ABK21562	7.32E-36
ACC23	3	histone h4	E. japonica	ACX50406	2.75E-29
ACC24	3	histone h4	E. japonica	ACX50406	2.01E-38
ACC25	2	40s ribosomal protein	S. tuberosum	ABA40465	1.04E-33
ACC26	2	60s ribosomal protein l23	T. aestivum	AAP80667	1.56E-61
ACC27	2	cytochrome P450 protein	C. lanatus	BAD26579	6.95E-25
ACC28	2	histone 2	V. vinifera	XP_002271506	2.52E-39
ACC29	2	histone 2	V. vinifera	XP_002271506	2.62E-39
ACC30	2	histone h2	G. max	ACU13572	9.67E-43
ACC31	2	histone h3	Z. mays	ACG25088	1.12E-06
ACC32	2	histone h3.3	S. salar	ACI68311	1.83E-25
ACC33	2	histone h4	E. japonica	ACX50406	1.25E-35
ACC34	2	hypothetical protein	V. vinifera	XP_002277580	2.19E-71
ACC35	2	hypothetical protein isoform 2	V. vinifera	XP_002269823	1.49E-07
ACC36	2	No hit			
ACC37	2	polyubiquitin	A. thaliana	AAL09741	1.77E-36
ACC38	2	ribosomal protein s27	V. vinifera	XP_002273119	9.67E-35
ACC39	2	tabacum cDNA clone mRNA^a^	N. tabacum	FS383363	1.04E-140
ACC40	2	tabacum cDNA clone mRNA^a^	N. tabacum	AM808955	1.55E-86
ACC41	2	tabacum cDNA clone mRNA^a^	N. tabacum	DW004611	1.12E-60
ACC42	2	ubiquitin extension protein	C. annuum	ABK42077	2.29E-69
ACC43	2	wox2 protein	Petunia × hybrida	ACA64094	1.28E-42
(b) Basal cell					
BCC01	62	NADH dehydrogenase subunit 7	Beta vulgaris	NP_064055	1.72E-58
BCC02	30	No hit			
BCC03	24	tabacum cDNA clone mRNA^a^	N. tabacum	FG626245	1.69E-73
BCC04	20	hypothetical protein	R. communis	XP_002524507	1.46E-06
BCC05	16	pathogenesis-related protein 10	S. lycopersicum	BAD95797	5.62E-11
BCC06	15	cacao cDNA clone mRNA^a^	T. cacao	CU503631	9.02E-45
BCC07	11	tabacum cDNA clone mRNA^a^	N. tabacum	AM836152	0.00E+00
BCC09	9	acyl-COA-binding protein	V. vinifera	XP_002263421	3.10E-25
BCC10	8	eca1 protein	V. vinifera	XP_002275198	1.16E-27
BCC12	7	embryo abundant methyltransferase	P. trichocarpa	XP_002308901	4.35E-75
BCC13	7	sugar transport protein 8	V. vinifera	XP_002277946	1.62E-73
BCC14	6	tomato cDNA clone mRNA^a^	L. esculentum	BW690013	5.37E-53
BCC15	5	tabacum cDNA clone mRNA^a^	N. tabacum	FS398978	2.11E-162
BCC16	4	tabacum cDNA clone mRNA^a^	N. tabacum	FS395950	3.79E-78
BCC17	4	tabacum cDNA clone mRNA^a^	N. tabacum	FG623980	2.54E-102
BCC18	4	glutathione s-transferase	N. tabacum	P25317	4.85E-73
BCC19	4	nucleoside diphosphate kinase	N. tabacum	Q56E62	7.50E-11
BCC20	4	putative glucosyltransferase	L. esculentum	AAL92461	3.49E-37
BCC21	4	hypothetical protein	P. trichocarpa	XP_002330403	1.51E-22
BCC23	3	hypothetical protein	V. vinifera	XP_002284032	2.71E-21
BCC24	3	hypothetical protein	V. vinifera	XP_002284032	2.10E-21
BCC25	3	defender against cell death 1	N.suaveolens × N.tabacum	BAB40808	1.06E-17
BCC27	3	histone h3.3B isoform 2	C. intestinalis	XP_002131754	7.29E-22
BCC28	3	pollen allergen	R. communis	XP_002517629	5.29E-15
BCC29	3	acyl-COA-binding protein	V. vinifera	XP_002263421	1.39E-25
BCC30	3	60s ribosomal protein l35a	G. max	ACU13467	1.01E-24
BCC31	2	hypothetical protein	Escherichia	ZP_04532941	3.73E-10
BCC32	2	tabacum cDNA clone mRNA^a^	N. tabacum	EB431770	2.73E-141
BCC33	2	tabacum cDNA clone mRNA^a^	N. tabacum	EH623820	1.86E-137
BCC34	2	60s ribosomal protein l29	L. esculentum	AAG49033	4.56E-16
BCC35	2	hypothetical protein	V. vinifera	CAN81050	1.07E-09
BCC37	2	histone h3	N. vectensis	XP_001632617	1.62E-26
BCC38	2	No hit			
BCC39	2	cell growth defect factor 2	V. vinifera	XP_002280550	6.60E-28
BCC40	2	tabacum cDNA clone mRNA^a^	N. tabacum	AM834148	3.30E-147
BCC41	2	40s ribosomal protein s19	S. tuberosum	ABB87116	3.90E-28
BCC42	2	DNA-directed RNA polymerase II	R. communis	XP_002522014	1.67E-26
BCC43	2	nucleoside diphosphate kinase	N. tabacum	Q56E62	1.21E-21
BCC44	2	hypothetical protein	R. communis	XP_002532758	1.73E-15
BCC45	2	tabacum cDNA clone mRNA^a^	N. tabacum	BP530929	4.41E-106
BCC46	2	No hit			
BCC47	2	histone h3	C. clemensi	ACO15671	7.43E-27
BCC48	2	tabacum cDNA clone mRNA^a^	N. tabacum	FS407739	2.26E-170

The contigs are sorted according to the number of ESTs.

^a^The results of BLASTN search.

**Figure 3:**
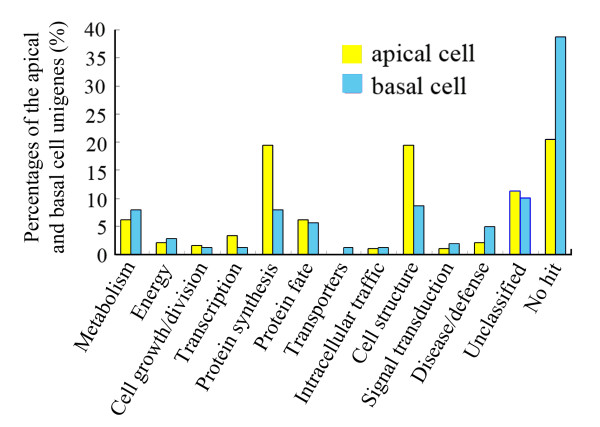
**Functional classification of differentially expressed transcripts in apical and basal cells of tobacco two-celled proembryo**. Percentages of the 167 apical and 131 basal cell unigenes are represented by yellow and blue bars, respectively.

According to BLAST annotation, the functional role for each transcript is assigned on the basis of sequence similarity to proteins with known functions in GenBank. The classification results according to 13 major functional categories are shown in Figure 3, and the detailed classification of each unigenes is listed in Additional file 2. Transcripts related to protein synthesis and cell structure represent the largest group of transcripts with known function in the apical cell, and both compose of 20.36% of the apical cell unigenes. In the basal cell, transcripts related to protein synthesis and cell structure also are present with high percentage, followed by related to metabolism, protein fate and disease/defense. The transcripts related to metabolism and disease/defense compose of 8.40% and 5.34% of the total basal cell unigenes, respectively. Besides, 21.56% apical cell and 40.46% basal cell transcripts show no significant homology to public databases (Figure 3). This suggests that the apical and basal cell libraries in tobacco are highly effective for identifying transcripts that putatively encode novel proteins.

The subtracted apical and basal cell cDNA libraries provide a resource for identifying genes that involved in embryogenesis. To test the speculation, a BLASTX search was performed to identify genes involved in *Arabidopsis *embryo development in the subtracted cDNA libraries. The result of search (with cutoff e-value of ≤10^-5^) showed that 12 transcripts in our libraries encoded putative homologies involved in embryo development, with 11 of them similar to *EMB *genes (Table 2). Based on sequence conservation, the biological functions of these tobacco homologies are possibly similar to those of *Arabidopsis *genes, however, the illustration of their detailed function in tobacco embryogenesis still need further research.

**Table 2:** Putative tobacco homologies of Arabidopsis genes involved in normal embryo development

ID	Chromosome Locus	Gene Symbol	BLAST e-Value/% Identity/%% Similarity	Predicted function
ACC26	At3g04400	EMB 2171	1e-80 96% 100%	Ribosomal Protein L17/L23
ACC37	At3g52590	EMB 2167	1e-41 98% 100%	Ubiquitin Fused to Ribosomal Protein L40
ACC43	At5g59340	WOX2	4e-16 35% 46%	WUSCHEL RELATED HOMEOBOX 2
AC146C	At5g15540	EMB 2773	2e-07 35% 40%	Adherin sister-chromatid cohesion 2
AC161C	At3g52380	PDE 322	1e-15 33% 61%	Chloroplast RNA Binding Protein
AC327C	At2g18510	EMB 2444	2e-10 36% 52%	Spliceosome Associated Protein
AC336C	At2g04030	EMB 1956	4e-49 55% 75%	Heat Shock Protein (Hsp90)
AC338C	At3g11670	DGD 1	4e-10 50% 70%	Digalactosyl Diacylglycerol Synthase
AC349C	At3g52590	EMB 2167	1e-12 39% 59%	Ubiquitin Fused to Ribosomal Protein L40
AC364C	At3g11940	AML 1	2e-87 88% 95%	Ribosomal Protein S5
BC101C	At5g10480	PAS 2	7e-31 75% 87%	Protein Tyrosine Phosphatase Like
BC117C	At3g54010	PAS 1	2e-08 33% 47%	Immunophilin-like FK506 Binding Protein

These sequences were identified by BLASTX search against Arabidopsis sequence database of genes involved in embryogenesis.

### Validation of the differential expression in zygote and its two daughter cells

**Figure 4:**
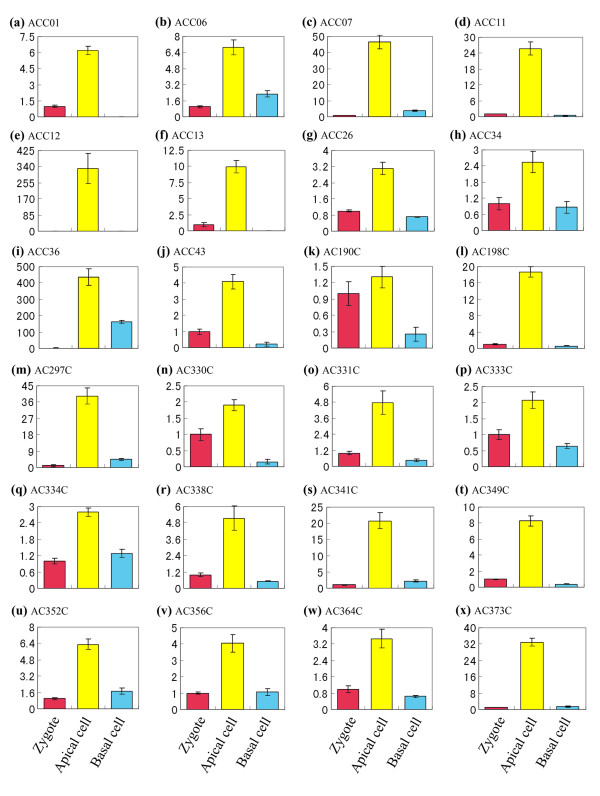
**Real-time PCR analysis for transcripts of the apical cell cDNA library in zygote and its two daughter cells**. The transcripts are indicated by library ID number. cDNAs synthesized from zygotes, apical cells and basal cells are used as template for PCR amplification. The expression level in zygote is set as reference with the default value 1.

**Figure 5:**
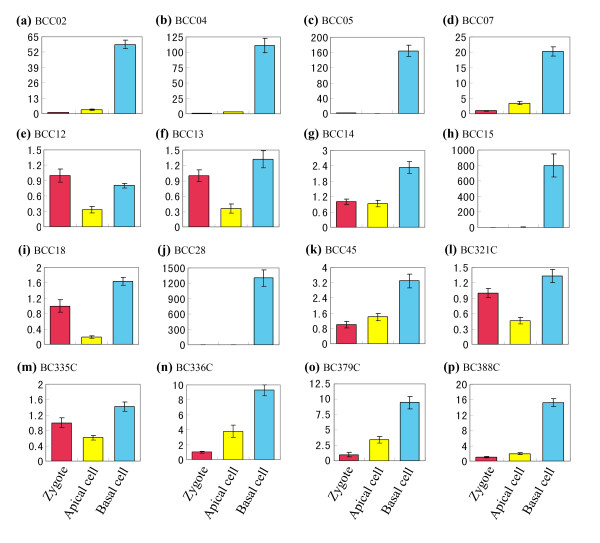
**Real-time PCR analysis for transcripts of the basal cell cDNA library in zygote, and its two daughter cells**. The transcripts are indicated by library ID number. cDNAs synthesized from zygotes, apical cells and basal cells are used as template for PCR amplification. The expression level in zygote is set as reference with the default value 1.

To validate the differential expression revealed by macroarray, we used quantitative real-time PCR to detect the differential expression of 40 candidate transcripts (Figures 4 and 5). As no tested gene displayed consistent expression across the samples, none of these genes was selected as internal control. Therefore, we taken the expression levels of candidate genes in zygote as reference with the default value 1, and then calculated the relative expression levels in the apical and basal cells. The results showed that all the tested transcripts displayed expression differences over two fold between the apical and basal cells. Among these tested transcripts, three transcripts (ACC01, ACC12 and ACC13) were expressed specifically (with expression difference more than 100 fold) in the apical cells (Figure 4a, e, f), while other three (BCC05, BCC15 and BCC28) in the basal cells (Figure 5c, h, j).

Combining the zygotic expression of transcripts with the expression in apical and basal cells, two different expression patterns were observed in the examined transcripts: (i) specifically expressed only in the apical cell (Figure 4d, e, l, m, x) or in basal cell (Figure 5a-c, h, j); (ii) expressed in zygote, and subsequently predominantly in the apical cell (Figure 4k, n, p) or in the basal cell (Figure 5e, f, l, m). It's very interesting that about one third of the tested transcripts showed only negligible signals in the zygote, while greatly enhanced the expression in one of the daughter cells after zygotic division, suggesting an mechanism controlling specific gene activation in the apical and basal cells for early embryogenesis.

### Whole mount *in situ *hybridization of the isolated zygote and two-celled proembryos

**Figure 6:**
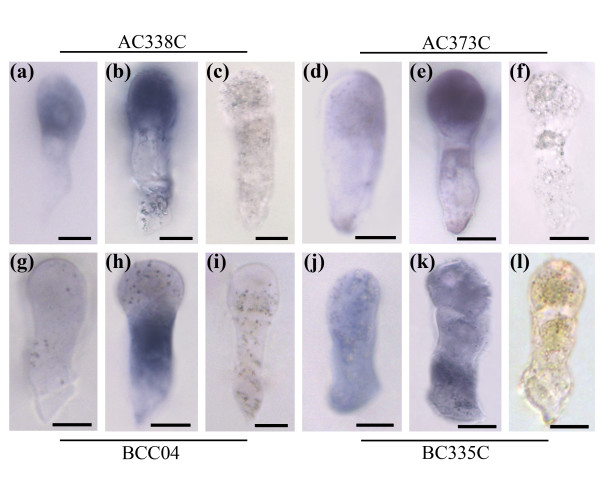
***In situ *hybridization analysis of transcripts with differential expression in apical and basal cells**. Isolated zygotes and two-celled proembryos were hybridized with antisense (a, b, d, e, g, h, j, k) or sense (c, f, i, l) digoxigenin-labelled RNA probes for AC338C (a-c); AC373C (d-f); BCC04 (g-i); BC335C (j-l) transcripts. Scale bar in all pictures = 10 μm.

We performed whole mount *in situ *hybridization in the isolated zygote at the elongated stage and the two-celled proembryo as an additional stringent test to validate our real-time PCR results of differential gene expression in zygote and its two daughter cells. We detected the expression patterns of four candidate genes from apical and basal cell libraries. The results confirmed the expression patterns of SSH identified genes validated by real-time PCR (Figure 6). The signal of AC338C transcript was detected predominantly in the apical pole of zygote and the apical cell of the two-celled proembyo (Figure 6a, b). The expression of AC373C was undetectable in zygote, but specifically initiated expression in the apical daughter cell (Figure 6d, e). For the basal cell transcripts, the expression of BCC04 was presented exclusively in the basal daughter cell, but not in the zygote and its apical daughter cell (Figure 6g, h). In contrast, BC335C transcript displays weak signal in the zygote, while predominant expression in the basal daughter cell (Figure 6j, k). All the two-celled proembryo samples hybridized with sense probes showed no signal (Figure 6c, f, i, l). These expression data are consistent with the real-time PCR results (Figures 4 and 5).

### Expression analysis of candidate genes in different organs and tissues

**Figure 7:**
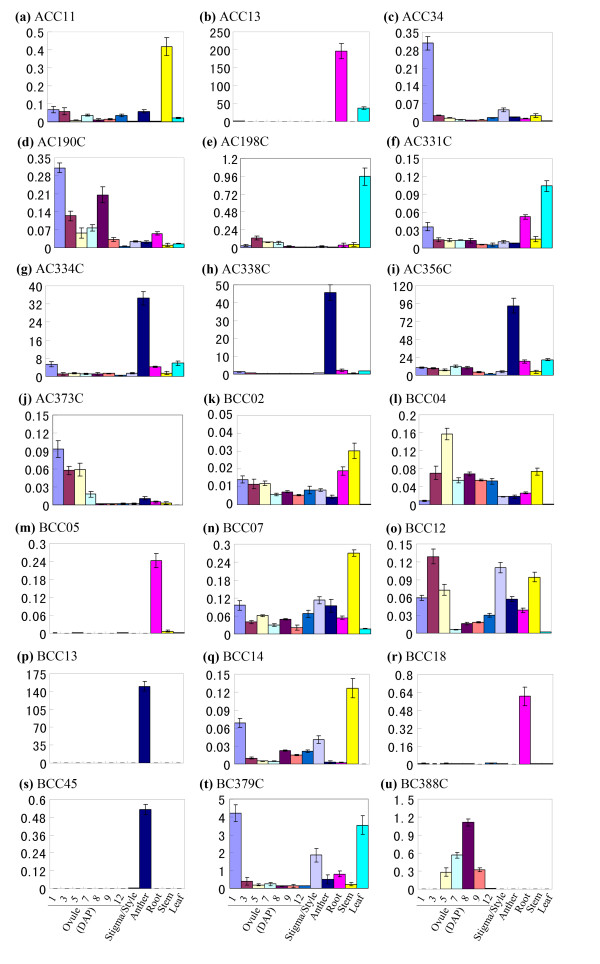
**Expression analysis for transcripts differentially expressed in the apical and basal cells by quantitative real-time PCR**. All expression presented is relative to that of the reference gene *GAPD*.

We analyzed the expression of the validated differential transcripts in different organs and tissues of tobacco (Figure 7). All of the detected transcripts displayed differential expression in the tested organs and tissues. The four transcripts (ACC34, AC190C, AC373C and BC379C) were expressed at high levels in 1 day after pollination (DAP) ovule and then gradually decreased along with the development of ovules (Figure 7c, d, j, t). Among these transcripts, expression of ACC34 and BC379C declined immediately after fertilization, suggesting their possible roles in ovule development and fertilization. However, the expressions of two other transcripts BCC04 and BC388C were firstly up-regulated after fertilization and then gradually decreased during the maturation of seed. Apart from the expression in ovules, three transcripts (ACC13, BCC05 and BCC18) displayed predominant expression in root, three transcripts (ACC11, BCC07 and BCC14) in stem, and three transcripts (AC198C, AC331C and BC379C) in leaf, with all nine transcripts showing moderate to weak signal in other organs (Figure 7). Interestingly, the expressions of five transcripts AC334C, AC338C, AC356C, BCC13 and BCC45 were abundant in anthers but scarce in ovules and vegetative tissues. Therefore, these results indicate that the apical and basal cell transcripts may play more general roles during plant growth and development.

## Discussion

### The apical and basal cells of tobacco two-celled proembryo possess distinct transcriptional profiles

**Figure 8:**
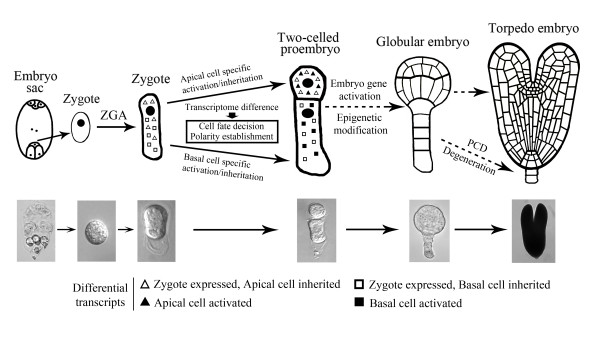
**Mechanism profile of embryo apical-basal axis formation and cell fate decision during the early embryogenesis of tobacco**. The bright field images display the truly representative stages of tobacco embryo. ZGA: zygotic gene activation, PCD: programmed cell death.

In dicotyledonous plant tobacco, cell division of zygotic embryo follows the same settled pattern as classical model plant *Arabidopsis*. The zygote firstly undergoes an asymmetric transverse division to shape a two-celled proembryo with a small apical cell and a large basal cell. Up to date, though the two daughter cells from zygote display a lot of differences in morphological characters, the internal molecular differences still remain unknown. Some presented evidences indicate that the two cells could be distinguished by their different gene expression. In scarlet runner bean, *G564 *and *C541 *mRNAs are only present in the two basal cell descendants of proembryos at the four-cell stage [37]. In *Arabidopsis*, some members of *WUSCHEL-RELATED HOMEOBOX *(*WOX*) and *PIN-FORMED *(*PIN*) genes families as well as *Arabidopsis thaliana MERISTEM LAYER 1 *(*ATML1*) gene all displayed specific expression in one of the daughter cells after zygote division [38-40]. Here we for the first time, carried out a comparative transcriptome analysis on the two cells, and successfully identified a lot of differential transcripts in the apical and basal cells. Transcript expression validation by quantitative real time PCR and *ISH *technique demonstrated that these isolated transcripts displayed a polar distribution in the two-celled proembryos. During these genes, the expression of transcript AC338C appeared in the apical pole of zygotes, and further enhanced in the apical cells (Figure 6). Moreover, the transcripts ACC07, ACC11, ACC12, AC198C, AC297C, AC341C and AC373C were specifically initiated to express in the apical cells, while transcripts BCC02, BCC04, BCC05, BCC15 and BCC28 in the basal cells (Figures 4 and 5). Considering some negligible signals, several transcripts were identified as apical or basal cell specific genes (Figures 4a, e, f and 5c, h, j). These differences reveal that the two daughter cells possess significantly distinct transcriptional profiles after the first zygotic division (Figure 8).

Zygotic gene activation (ZGA) is a critical event during early embryogenesis, which means the transfer of development control from parents to zygote and embryo. Some evidences substantiate the assumption that ZGA in higher plants occurs shortly after fertilization [6,33,34,41]. In our study, some differential transcripts were expressed in zygote and then only in one of the daughter cells, suggesting the possibility that these transcripts were specifically inherited by the apical or basal daughter cell (Figure 8). The other tested transcripts just displayed weak or negligible expression in zygote, but strong in the apical or basal daughter cell. The results indicate that apart from ZGA, further gene activation in early embryogenesis may also happen in the apical and basal cells, respectively (Figure 8). These cell specific transcript inheritation and activation may lead to the transcriptome differences in the apical and basal cells.

### Some candidate genes from the apical and basal cells play potential roles in embryo and post-embryo development

It seems that some of the 299 transcripts encode proteins required for gamete and early embryo development based on the homology search against *Arabidopsis *and rice. As shown in Table 2, 12 transcripts encode homologies of the genes involved in *Arabidopsis *embryo and seed development, such as *PASTICCINO1/2 *(*PAS1/2*) and *WOX2*. In *Arabidopsis*, *WOX2 and WOX8 *genes are expressed complementarily in the apical and basal cells in a lineage-specific manner and regulate respective cell fate decision during early embryogenesis [39,42]. Our results show that the tobacco homology of *WOX2 *(ACC43) is also predominantly expressed in the apical cell of two-celled proembryo. Another apical cell transcript (ACC12), which encodes a tobacco lipid transfer protein, showed specific expression activation in the apical cells. In *Arabidopsis *embryogenesis, lipid transfer protein gene (*AtLTP1*) showed a specific position expression in the embryo, with transcript accumulation exclusively in the protodermal cells of the globular embryos and in the cotyledons and the upper end of hypocotyl in late stage of embryos [43].

In the basal cell transcripts, BCC12 transcript, a putative embryo abundant methyltransferase, displayed predominant expression in zygote and its basal daughter cell (Figure 5). In mouse embryogenesis, histone arginine methylation mediated by arginine methyltransferase 1 (CARM1) contributes to cell fate decision in the four-cell-stage of embryo [44]. Moreover, the mutant analysis for *Arabidopsis METHYLTRANSFERASE1 *(*MET1*) and *CHROMOMETHYLASE3 *(*CMT3*) gene revels that DNA methylation is critical for the regulation of cell fate decision during early embryogenesis [45]. Besides, the basal cell transcript BCC39 encodes a tobacco homology of the cell growth defect factor 2 (Cdf2) in *Arabidopsis *[46], and the overexpression of Cdf2 caused Bax-like lethality in yeast [47]. Bax is a mammalian proapoptotic member of the Bcl-2 family, and the overexpression of Bax in *Arabidopsis *mesophyll protoplasts resulted in cytological apoptosis characteristics [48]. Therefore, such gene may involve in the programmed cell death (PCD) mediated degeneration of the future suspensor (Figure 8).

In our study, tissue expression analyses also show that several transcripts are abundant in the different stages of ovules, but barely detectable in vegetative tissues, indicating their possible functions in embryo and ovule development as well as seed formation, such as transcripts ACC34, AC190C, AC373C and BC388C (Figure 7). Besides expression in ovules, five differential transcripts (AC334C, AC338C, AC356C, BCC13 and BCC45) in the apical and basal cells displayed predominant expression in mature anthers. In *Arabidopsis*, the mitogen-activated protein kinase gene *YDA *functions in the process of zygote elongation and subsequent cell division, and regulates the first cell fate decision of the basal lineage [6]. Recently, the study reveals that the *SHORT SUSPENSOR *(*SSP*) transcripts accumulate in mature pollens, and then are delivered via the sperm cells to zygote, where SSP protein is produced to activate *YDA*-dependent signalling [41]. On one hand, our anther expressed transcripts may play roles in anther development, and on the other hand, it is reasonable to speculation that these transcripts are transferred to zygote via sperm cells and regulate the subsequent embryo development. Furthermore, another three transcripts (ACC13, BCC05, BCC18) show preponderant expression in tobacco roots (Figure 7). It's well known that auxin is important for pattern formation in embryo and root development [49,50]. In our study, one root expressed transcript BCC18 encodes an auxin induced parA protein in tobacco [51], suggesting that this protein may involve in auxin regulated embryo differentiation and subsequent root formation. Further research of these candidate genes in this study will contribute to elucidate the regulation mechanism of early embryo polarity establishment and pattern formation as well as succedent organ development in higher plants.

## Conclusions

Here we first established a procedure for isolating the live apical and basal cells of tobacco two-celled proembryo just after the first zygotic cell division *in vivo*. In dicotyledon plant, for the first time, we carried out a global investigation to the transcription profiles of the apical and basal cells *in vivo *by applying SSH technique coupled with macroarray hybridization. Further validation by quantitative RT-PCR and *ISH *technique showed that some differential and specific transcripts in the apical and basal cells of two-celled proembryos were successfully isolated, and the differential and specific expression of these transcripts revealed that the transcription compositions in the apical and basal cells are significantly distinct. Transcripts with specific expression in the apical and basal cells provide useful markers for research on the early embryogenesis. Some identified genes specifically expressed in the ovules, suggesting close relation to specific events of the embryo and seed development. Therefore, functional analysis of these genes will promote promising research on molecular mechanism of embryogenesis and seed development.

## Methods

### Accession numbers

All 797 EST sequences in the study (library ID AC001C- 385C and BC001C-412C) were deposited in GenBank with accession numbers from GT270790 to GT271586.

### Isolation of the zygote and the apical and basal cells from the two-celled proembryo

Tobacco (*Nicotiana tabacum cv. *SR1) plants were grown in a greenhouse with a photoperiod of 16 h light/8 h dark at 25-27°C. The elongated zygotes and the two-celled proembryos were isolated respectively from ovules at 84 and 108 h after pollination (HAP) according to the method of Qin *et al. *[12]. The isolated two-celled proembryos were collected into a droplet of 13% (w/v) sterile mannitol solution (pH 5.7) with a micropipette. To avoid the confusion of apical cells and basal cells from different two-celled proembryos, each proembryo was then transferred into an individual droplet of mannitol solution containing 1% cellulase onozuka-R10 (Yakult), 0.5% pectinase (Sigma), 1% hemicellulase (Sigma) and 0.5% snailase (Sigma) for enzymolysis. The two-celled proembryos were incubated in the enzyme solution for 10-15 min at 25°C. By gently sucking and spitting with a micropipette, a pair of protoplasts with a small apical cell and a large basal cell was separated. The two kinds of protoplasts were respectively collected into fresh 13% (w/v) mannitol droplets and washed twice, then transferred into the lysis/binding buffer and immediately frozen in liquid nitrogen. The viability of isolated protoplasts was detected using 50 mg/L fluorescein diacetate (FDA; Sigma) staining. Two transcription inhibitors, actinomycin D (50 mg/L, Sigma) and cordycepin (100 mg/L, Sigma), proven effective in suppressing the expression of stress-inducible genes [52], were added to all solutions in the process of cell isolation.

### RNA isolation of the zygote, apical and basal cells and cDNA synthesis

For each independent cDNA synthesis, RNA from about two hundred zygotes, three hundred apical cells or basal cells were respectively extracted using the Absolutely RNA Nanoprep Kit (Stratagene) according to the manufacturer's instructions. Then cDNA was synthesized and amplified using a Super SMART PCR cDNA Synthesis Kit (Clontech). The optimal LD-PCR cycle number was determined empirically to ensure the cDNA remained in the exponential phase of amplification. Approximately 100 ng synthesized cDNA was analyzed on a 1.2% agarose gel alongside 100 ng 1 kb DNA ladder. Then, the amplified cDNAs of the apical and basal cells were used for SSH and templates for gene-specific expression analysis.

### Suppression subtractive hybridization

The generation of forward- and reverse-subtracted cDNA and unsubtracted control cDNA from the apical and basal cells was performed using the PCR-Select cDNA Subtraction Kit (Clontech) following the manufacturer's instructions. Two rounds of hybridization and PCR amplification were performed to enrich the differentially expressed sequences, with 30 fold excess of the driver cDNA to select against for the first round subtraction and 2.5 fold for the second round subtraction. The subtracted apical and basal cell cDNAs were purified using QIAquick PCR Purification Kit (Qiagen), cloned with the pGEM-T Easy Vector System (Promega) and then transformed into *Escherichia coli DH5α *cell. The transformed bacteria were plated onto LB agar plates containing ampicillin, X-gal and IPTG. For constructing the subtracted apical and basal cell libraries, 4032 and 3300 recombinant white colonies were picked respectively, and cultured in 80 μl LB freezing medium with ampicillin in 384-well microtitre plates. After overnight culture, the plates were stored at -80°C for membrane printing.

### Colony and cDNA macroarray preparation

Colony and cDNA macroarrays were used respectively for the first and second round screenings. For the colony macroarray, all selected clones from subtracted libraries were printed onto Hybond-N+ nylon membranes (Amersham Biosciences) using the Genetix QPix 2 Colony Picker Systems (Genetix Ltd). The nylon membranes were then placed onto LB agar plates with ampicillin and incubated at 37°C overnight. For the succedent cDNA macroarray, the first-round validated colonies were picked out for the second round screening with plasmids. The plasmids were isolated from overnight-grown bacterial cultures using a standard alkaline lysis protocol with SDS in 96-well format and then printed onto Hybond-N+ nylon membranes. All the macroarray membranes were treated following the user manuals, with DNA crosslinked to membranes by baking at 80°C for 2 h, and then were stored at -20°C for differential screenings.

### Preparation of probes and cDNA differential screening

The probe labeling and macroarray hybridization were carried out using the PCR-select Differential Screening Kit (Clontech). The membranes were prehybridized for 40-60 min at 72°C, and then hybridized with the radioactive probes at 72°C overnight. The hybridized membranes were washed in 2 × SSC and 0.5% SDS for 4 × 20 min, in 0.2 × SSC and 0.5% SDS for 2 × 20 min at 68°C, and then exposed to PhosphorImager screens (Amersham Biosciences) for 24 hours. Images were acquired by scanning the membranes with a Typhoon 9210 scanner (Amersham Biosciences), and data analysis was performed using ArrayVision 8.0 software (Amersham Biosciences). The clones showing the most marked differential expression were selected for sequencing.

### Sequence and bioinformatics analysis

The differentially expressed clones identified by screening were picked for sequencing with ABI3730 machines (Applied Biosystems). The vector and adaptor sequences were trimmed using Vector NTI Advance 9 software (Informax). After pre-processing, the expressed sequence tags (ESTs) were clustered and assembled into contigs using online tool EGassembler (http://egassembler.hgc.jp/; [53]). The assembled consensus sequences of contigs and valid ESTs were used as a query for BLASTN and BLASTX searches http://blast.ncbi.nlm.nih.gov/Blast.cgi, with significance threshold score >115, expected value <e^-25 ^for BLASTN, and an e-value of <e^-5^, score > 50 for BLASTX. Transcripts encoding proteins of known functions were manually categorized into the functional classification described by Bevan *et al. *[54], with reference to the scarlet runner bean (SRB) embryonic EST project (http://www.mcdb.ucla.edu/Research/ Goldberg).

### Gene expression analysis by quantitative real-time PCR

For expression analysis in the zygote and its two daughter cells, pre-amplified double-stranded cDNAs (ds cDNAs) using the Super SMART PCR cDNA synthesis kit were used. After purification and measurement, 20 ng of ds cDNA from each sample was used as template for real-time PCR analysis by SYBR-green fluorescence using the Rotor-Gene Q6000 system (Corbett Life Science). Cycling parameters were as follows: 94°C for 10 sec, 56°C for 20 sec, and 72°C for 30 sec. The cDNA samples used were independent from those of the SSH analysis and all expression patterns were confirmed by using two independent cDNA samples. For every examined gene, the expression levels in each sample relative to zygote were calculated.

For expression pattern analysis among different organs, the materials were taken as follows: root, stem and leaf from the one-month-old plants, anther and stigma/style from anthesis-stage flowers, 1 DAP (day after pollination ) ovules at the egg-celled stage, 3 DAP at the zygote, 5 DAP at early globular embryo, 7 DAP at late globular embryo, 8 DAP at heart-shaped embryo, 9 DAP at torpedo-shaped embryo and 12 DAP at cotyledon-staged embryo. Each reaction contained equal amount of sample cDNA, and the reaction was repeated at least twice. The constitutively expressed glyceraldehyde-3-phosphate dehydrogenase (*GAPD*) gene (Accession number AJ133422) was used as an internal standard. Primer pairs were all designed with Primer Premier Software (Premier Biosoft International) and listed in the Additional file 3.

### Whole mount *in situ *hybridization

Digoxigeninlabeled RNA probes were generated with the DIG RNA labeling kit (Roche) according to the manufacturer's instructions, and *in situ *hybridization was performed as described by Hejátko *et al*. [55], with modification of embedding the isolated zygotes and the two-celled proembryos in 12% polyacrylamide. The elongated zygotes and the two-celled proembryos were isolated respectively from ovules at 84 and 108 h after pollination (HAP). Gel pieces containing the zygotes and proembryos after hybridization were incubated in a 1:2000 Anti-DIG-Antibody (Roche), and transcripts were detected colorimetrically by the DIG nucleic acid detection kit (Roche). The images were digitally recorded with a BH2 microscope (Olympus) and the digital sight DS-U2 camera system (Nikon).

## Authors' contributions

TXH and MY contributed equally in the design and execution of the experiment, and participate in the analyses of the study. TXH drafted the manuscript. JZ conceived of the study, and participated in its design and coordination. All authors contributed to the revision of manuscripts, and approved the final manuscript.

## Supplementary Material

Additional file 1 **List of contig EST constitutions after sequence assembly**. Click here for file

Additional file 2 **Unigene information list of blast annotation results and function classification**. Click here for file

Additional file 3 **List of primers used for real-time PCR analysis of zygote, apical cell, basal cell, organ and tissue specific expression**. Click here for file
